# Bis(2-chloro-*N*,*N*-di­methyl­ethan-1-aminium) tetra­chlorido­cobaltate(II) and tetra­chlorido­zincate(II)

**DOI:** 10.1107/S2056989024003955

**Published:** 2024-05-10

**Authors:** Katelyn McGinness, Kim Minton, Katelyn White, Marcus R. Bond

**Affiliations:** a Department of Chemistry and Physics, Southeast Missouri State University, Cape Girardeau, MO 63701, USA; University of Missouri-Columbia, USA

**Keywords:** crystal structure, *gauche* effect, DFT calculation, disorder, mol­ecular switch

## Abstract

The few examples of structures containing the 2-chloro-*N*,*N*-di­methyl­ethan-1-aminium or 3-chloro-*N*,*N*-di­methyl­propan-1-aminium cations show a compet­ition between *gauche* and *anti* conformations for the chloro­alkyl chain. To explore further the conformational landscape of these cations, and their possible use as mol­ecular switches, the title salts, (C_4_H_11_ClN)_2_[CoCl_4_] and (C_4_H_11_ClN)_2_[ZnCl_4_], were prepared and structurally characterized. Details of both structures are in close agreement. The inorganic complex exhibits a slightly flattened tetra­hedral geometry that likely arises from bifurcated N—H hydrogen bonds from the organic cations. The alkyl chain of the cation is disordered between *gauche* and *anti* conformations with the *gauche* conformation occupancy refined to 0.707 (2) for the cobaltate. The *gauche* conformation places the terminal Cl atom at a tetra­hedral face of the inorganic complex with a contact distance of 3.7576 (9) Å to the Co^2+^ center. The *anti* conformation places the terminal Cl atom at a contact distance to a neighboring *anti* conformation terminal Cl atom that is ∼1 Å less than the sum of the van der Waals radii. Thus, if the *anti* conformation is present at a site, then the nearest neighbor must be *gauche.* DFT geometry optimizations indicate the *gauche* conformation is more stable *in vacuo* by 0.226 eV, which reduces to 0.0584 eV when calculated in a uniform dielectric. DFT geometry optimizations for the unprotonated mol­ecule indicate the *anti* conformation is stabilized by 0.0428 eV *in vacuo*, with no strongly preferred conformation in uniform dielectric, to provide support to the notion that this cation could function as a mol­ecular switch *via* deprotonation.

## Abstract 2

The competition between *gauche* and *anti* conformations in 2-chloro­ethyl- or 3-chloro­propyldi­methyl­ammonium cations is investigated for the title tetra­chloro­metallate salts in which the alkyl chain is found to disordered with the *gauche* conformation dominant.

## 1. Chemical context

The recently published structure of 3-chloro-*N*,*N*-di­methyl­propan-1-aminium chloride reported the conformation of the mol­ecular cation (henceforth 3CLPA^+^) as *gauche* for the terminal Cl atom (Bond & Silwal, 2023 ▸). DFT geometry optimizations *in vacuo* indicate that the *gauche* conformation is more stable than *anti* for the mol­ecular cation, as well as for the 2-chloro-*N*,*N*-di­methyl­ethan-1-aminium analog (henceforth 2CLEA^+^). In both cases the mol­ecular cations appear to exhibit the *gauche* effect in which the bonding pair of a C—H bond β to the terminal halogen atom of the chain is donated to the anti­bonding orbital of the C—*X* bond (*X* = halogen) to stabilize the *gauche* conformation through hyperconjugation (Wolfe, 1972 ▸). A recent computational study of 1,2-dihalo­ethanes reports that this stabilization due to hyperconjugation is always present, but other energy contributions – such as steric inter­actions – are more important so that the *gauche* effect is typically observed only with fluorine (Rodrigues Silva *et al.*, 2021 ▸). However, in the 3CLPA^+^ and 2CLEA^+^ cations the terminal Cl atom is closer to the formal center of positive charge when in the *gauche* conformation, which may provide an additional contribution to energetic stability.

We are inter­ested in investigating the competition between *gauche* and *anti* conformations for these mol­ecular cations in different chemical environments in order to explore their possible use as mol­ecular switches. If the *gauche* conformation is stabilized by inter­action with the positive charge center in the cation, then it is possible with loss of this inter­action through deprotonation that the *anti* conformation becomes more stable and would allow for a change in conformation by altering the degree of protonation. Here we report the structures of 2CLEA^+^ with the tetra­hedral complex anions CoCl_4_^2−^ and ZnCl_4_^2−^. 
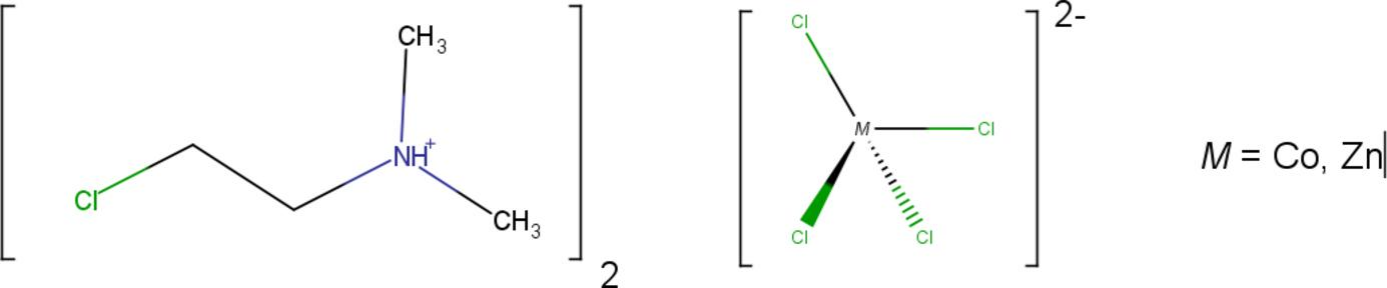


## 2. Structural commentary

The structures are isomorphous with unit-cell parameters in close agreement, *e.g.* unit-cell volumes agree within 2 s.u. Both tetra­hedral complexes occupy sites of twofold rotational symmetry with a slight flattening about the twofold axis to produce some Cl—*M*—Cl (*M* = Co, Zn) angles greater than 109.5°. *M*—Cl bond lengths range from 2.25–2.29 Å, in good agreement with average bond lengths of 2.27 (2) Å for CoCl_4_^2−^ and 2.27 (4) Å for ZnCl_4_^2−^ calculated from structures in the Cambridge Structural Database [512 and 960 hits, respectively; version 5.45 (November, 2023); Groom *et al.* (2016 ▸)].

**Figure 1:**
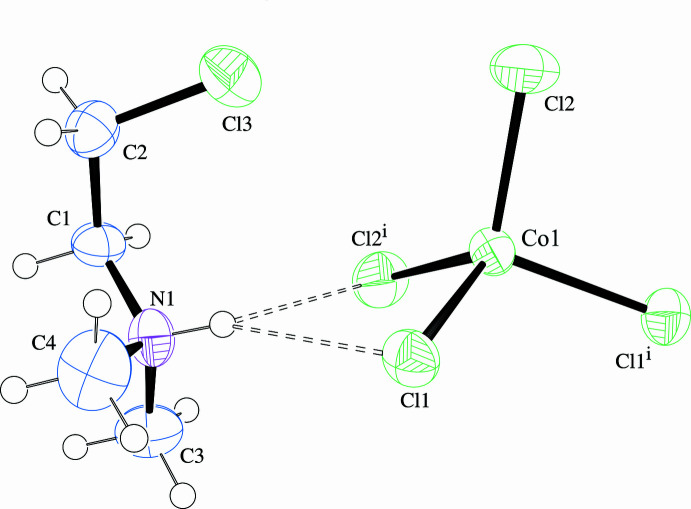
Displacement ellipsoid plot (50% level) of the organic cation and complex anion in (2CLEA^+^)_2_CoCl_4_ with labels for non-H atoms. The *gauche* conformation of the organic cation only is shown. H atoms are drawn as circles of arbitrary radii and N—H⋯Cl hydrogen bonding is represented by dashed lines.

**Figure 2:**
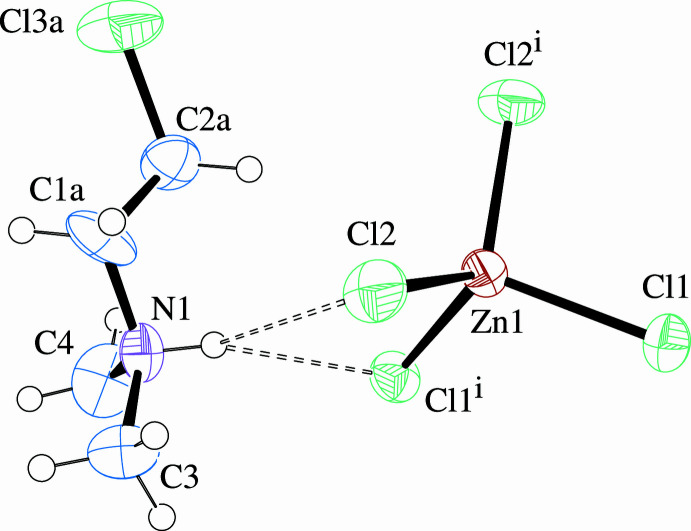
Displacement ellipsoid plot (50% level) of the organic cation and complex anion in (2CLEA^+^)_2_ZnCl_4_ with labels for non-H atoms. The *anti* conformation of the organic cation only is shown. H atoms are drawn as circles of arbitrary radii and N—H⋯Cl hydrogen bonding is represented by dashed lines.

Of greater inter­est is the organic cation, which exhibits disorder between the *gauche* [s.o.f = 0.707 (2) for CoCl_4_^2−^ and 0.697 (2) for ZnCl_4_^2−^] and *anti* conformations (the di­methyl­ammonium head group is ordered). The N1—C1—C2—Cl3 torsion angles are 61.6 (7)° in the CoCl_4_^2−^ and −61.3 (6)° in the ZnCl_4_^2−^ salts for the *gauche* conformation and, likewise, −179.7 (13) and 179.3 (9)°, respectively, for the *anti*. Bond lengths and angles within the cation correspond to expected values, disregarding small distortions that arise due to refinement of atoms of the disordered pair in close proximity. Displacement ellipsoid plots with labels for non-H atoms are presented in Fig. 1 ▸ for the CoCl_4_^2−^ salt showing only the *gauche* conformation of the organic cation, and in Fig. 2 ▸ for the ZnCl_4_^2−^ salt showing only the *anti* conformation. Bond lengths and angles for non-H atoms are presented in Table 1 ▸ for the CoCl_4_^2−^ salt, with only the *gauche* conformation values, and in Table 2 ▸ for the ZnCl_4_^2−^ salt, with only the *anti* conformation values.

**Figure 3:**
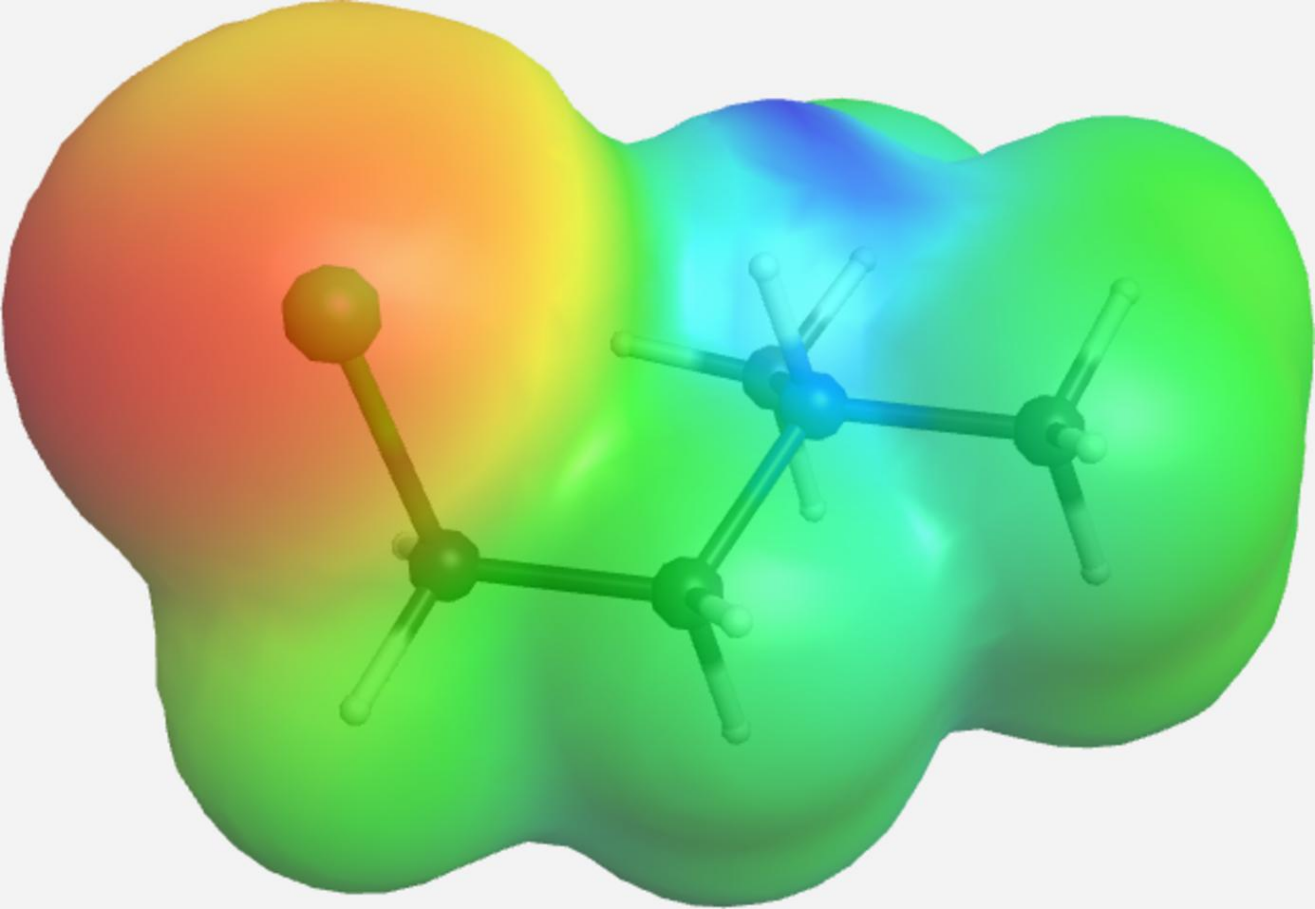
Electrostatic potential plot of the 2CLEA^+^ cation calculated in uniform dielectric for the *gauche* conformation with a ball-and-stick model of the optimized geometry shown within the electron density envelope. Red indicates regions of negative charge accumulation and blue regions of positive charge.

DFT geometry optimizations [B3LYP, 6311+G(d,p); *GAMESS* (Schmidt *et al.*, 1993 ▸)] *in vacuo* of 2CLEA^+^ yield an energy for the *gauche* conformation that is 0.226 eV less than the *anti* conformation for a 52.737° N—C—C—Cl torsion angle (0.228 eV less for a torsion angle of −52.738°). To approximate the ionic environment of the cation in the crystal, the optimizations were performed in a uniform dielectric constant of 78.4. This results in a reduction of *gauche* conformation stabilization to 0.0584 eV (torsion angle = 59.1°; by 0.0582 eV for torsion angle = −58.785°), but yields better agreement with observed torsion angle values. [Optimized torsion angles for the *anti* conformation with magnitudes of 173.858° (*in vacuo*) and 173.819° (dielectric) deviate slightly from observed values.] Similar optimizations for the unprotonated mol­ecule show both *gauche* conformations are *de*stabilized *in vacuo* (by 0.0412 eV for torsion angle = 66.377° and by 0.0428 eV for torsion angle = −64.735°). Energy differences for optimizations performed in uniform dielectric for the unprotonated mol­ecule are not as stark: *gauche* conformations are slightly stabilized by 0.00792 eV (−65.149°) or 0.00624 eV (64.459°). These results show promise of a switch from *gauche* to *anti via* deprotonation. An electrostatic potential plot of the *gauche* conformation from the uniform dielectric calculation is presented in Fig. 3 ▸.

## 3. Supra­molecular features

The di­methyl­ammonium headgroup forms an asymmetric, bifurcated hydrogen bond to Cl1 and Cl2 resulting in the most acute Cl—*M*—Cl angle in the complex anion – and a likely origin for the observed tetra­hedral flattening. Hydrogen-bond lengths and angles are presented in Tables 3 ▸ and 4 ▸ for the CoCl_4_^2−^ and ZnCl_4_^2−^ salts, respectively.

In the *gauche* conformation of the mol­ecular cation, the terminal Cl atom is placed slightly offset from the center of the tetra­hedral face defined by Cl1, Cl2, and Cl2^i^ [symmetry code: (i) 1 − *x*, *y*, 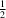 − *z*]. This places the terminal Cl atom at a distance of 3.7576 (9) Å from the Co^2+^ center [3.7690 (10) Å for Zn^2+^] with the shortest Cl⋯Cl contact distance [Cl2⋯Cl3 = 3.4293 (11) Å for CoCl_4_^2−^; 3.4237 (11) Å for ZnCl_4_^2−^] slightly less than 3.50 Å – the sum of the van der Waals radii. In the *anti* conformation, a methyl­ene H atom from the carbon α to the terminal Cl atom is instead directed at this face and forms contact distances of 3.2–3.3 Å to the Cl atoms. Meanwhile, the terminal Cl atom now forms a short contact [Cl3*A*⋯Cl3*A* ^ii^ = 2.588 (4) Å, Cl3*A*⋯Cl3*A* ^iii^ = 2.568 (4) Å; symmetry codes: (ii) 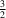 − *x*, 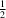 − *y*, 1 − *z*, for CoCl_4_^2−^; (iii) 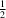 − *x*, 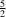 − *y*, −*z*, for ZnCl_4_^2−^] with a terminal *anti* conformation Cl atom in the nearest neighbor that is ∼1 Å less than the sum of the van der Waals radii. Hence, any *anti* conformation mol­ecular cation must have a *gauche* conformation cation as a nearest neighbor. This provides another driver for the dominance of the *gauche* conformation in these structures.

**Figure 4:**
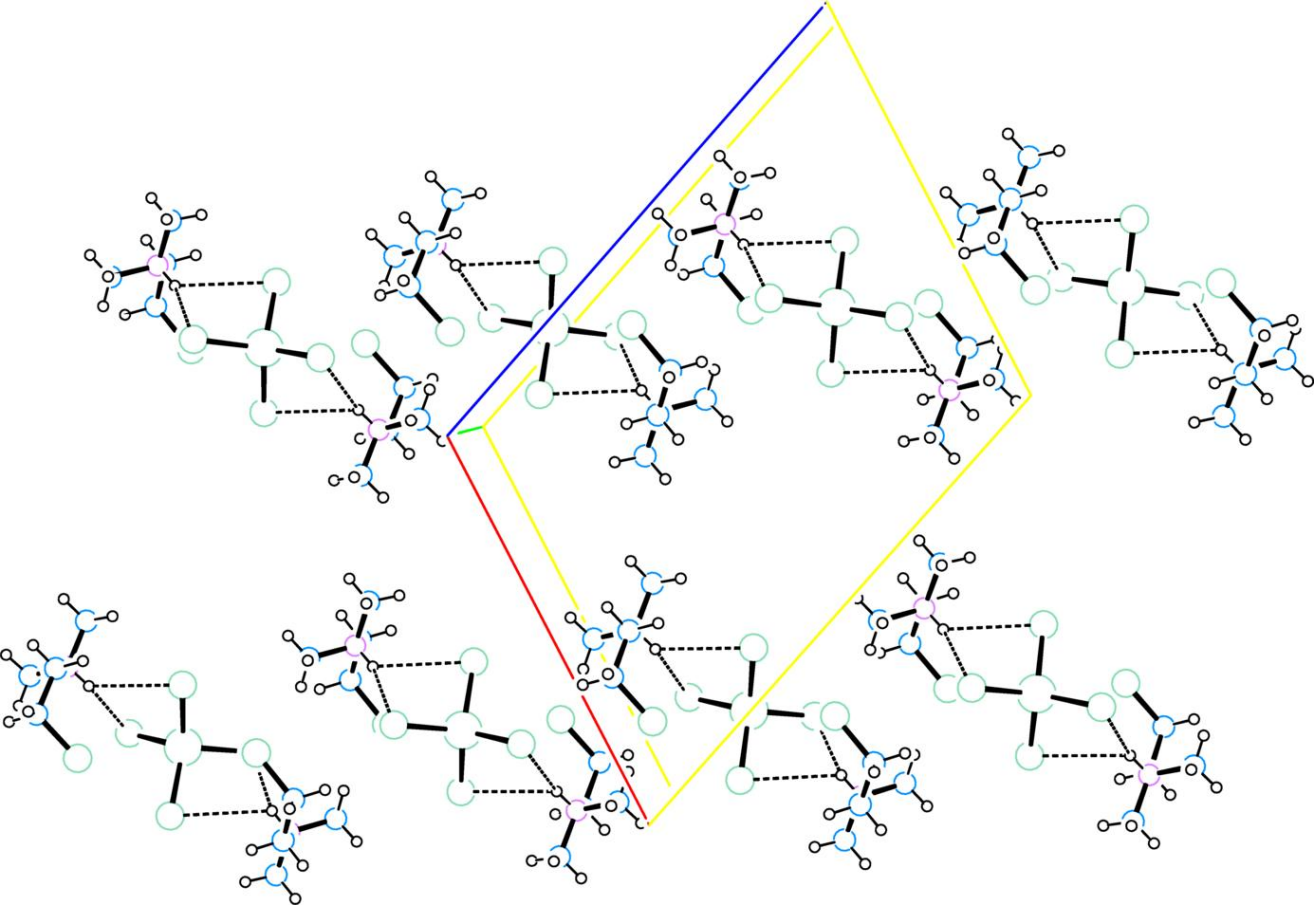
Layer packing diagram viewed down the *b* axis for (2CLEA^+^)_2_CoCl_4_ (*gauche* conformation only) that depicts portions of two rows of formula units along (101) that form a layer in the *ac* plane. The *a* axis slants down and to the right, the *c* axis slants up and to the right. Atoms are drawn as circles of arbitrary radii and N—H⋯Cl hydrogen bonding is represented by dashed lines

**Figure 5:**
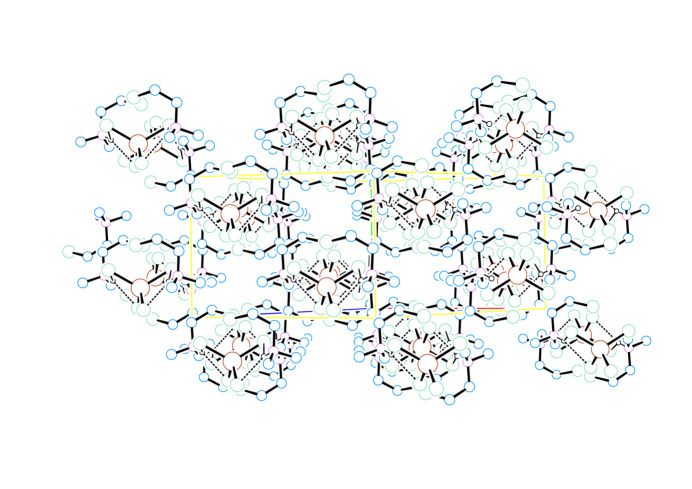
Unit-cell packing diagram for (2CLEA^+^)_2_ZnCl_4_ viewed down (101) showing the stacking of four of the layers presented in Fig. 4 ▸ with the *b* axis vertical. H atoms are omitted for clarity (except for N—H), atoms are drawn as circles of arbitrary radii, and N—H⋯Cl hydrogen bonding is represented by dashed lines.

The three-dimensional packing can be described starting with parallel rows of hydrogen-bonded formula units along (101) arranged into layers in the *ac* plane, as shown in the layer packing diagram of Fig. 4 ▸. Rows in neighboring layers nest between the rows of a given layer, with neighboring layers related by the *C*-centering translation, as shown in the unit-cell packing diagram of Fig. 5 ▸.

## 4. Database survey

Structural results for 2CLEA^+^ or 3CLPA^+^ cations have been sparsely reported. A survey of the Cambridge Structural Database (version 5.45, November, 2023; Groom *et al.*, 2016 ▸). yields only two known prior examples of structures containing 2CLEA^+^: an (Mo_2_O_2_Cl_8_)^2−^ salt (CSD refcode POSWAX; Marchetti *et al.*, 2015 ▸), in which the *gauche* conformation is found, and a chloride salt (CSD refcode: URORUR; Muller *et al.*, 2021 ▸) where the *anti* conformation is found, albeit with a disordered alkyl chain. For 3CLPA^+^, besides the aforementioned chloride salt there is one other structure containing 3-chloro-*N*,*N*-propan-1-amine as a ligand in a di(μ-hydrido) dialuminium complex (CSD refcode: NIGGOZ; Andrews *et al.*, 1997 ▸) where the *gauche* conformation is also found. No structures containing the unprotonated or uncoordinated mol­ecules have been reported. There are also no reported structures for the longer chain chloro­butyl or chloro­pentyl analogs.

## 5. Synthesis and crystallization

Both compounds were prepared by dissolving 2-chloro-*N*,*N*-di­methyl­ethan-1-aminium chloride with 1.00 g of CoCl_2_·6H_2_O or ZnCl_2_ in a 2:1 molar ratio in water. The solutions were acidified with concentrated HCl(aq) to yield ∼6 *M* HCl and produce a definite blue color in the cobalt(II) solution. The solutions were evaporated to a syrup with the syrup redissolved in ethanol to yield crystals of the title compounds upon further evaporation.

## 6. Refinement

Crystal data, data collection, and structure refinement details are summarized in Table 5 ▸. For both compounds, initial structure solution identified positions of all non-H atoms except those of the *anti* conformation. Prominent electron density difference map peaks then identified atoms of the *anti* conformation. Common site occupation factors for each conformation were refined with the constraint that their sum equal 1.0. H-atom positions were visible on the electron density difference map, but were calculated and refined using a riding model for those bound to C with isotropic displacement parameters set to 1.2 or 1.5×*U_iso_* of the parent atom for methyl­ene or methyl H atoms, respectively. The H atom bound to N was freely refined to a reasonable N—H bond length and the N1—C1*A* distance was constrained to a chemically reasonable distance (1.50±0.01 Å) using the DFIX command in *SHELX*.

Low angle reflections (four for CoCl_4_^2−^ and one for ZnCl_4_^2−^ salts) with *F_o_* ^2^<<*F_c_* ^2^ were assumed to be blocked by the beam catcher and were omitted from the refinement. For the ZnCl_4_^2−^ structure, *APEX3* control software suggested a data-collection strategy to θ*max* = 36°. However data analysis (*WinGX 2021.3*; Farrugia, 2012 ▸) indicated <*I*/σ> less than 2.0 for reflections beyond θ = 30.6°. Thus reflection data beyond θ = 31° were omitted from the final refinement.

## Supplementary Material

Crystal structure: contains datablock(s) global, cobaltate, zincate. DOI: 10.1107/S2056989024003955/ev2006sup1.cif

Structure factors: contains datablock(s) cobaltate. DOI: 10.1107/S2056989024003955/ev2006cobaltatesup2.hkl

Structure factors: contains datablock(s) zincate. DOI: 10.1107/S2056989024003955/ev2006zincatesup3.hkl

CCDC references: 2352359, 2352358

Additional supporting information:  crystallographic information; 3D view; checkCIF report

## Figures and Tables

**Table 1:** Selected geometric parameters (Å, °) for cobaltate[Chem scheme1]

Co1—Cl1	2.2873 (6)	N1—C1	1.509 (5)
Co1—Cl2	2.2618 (6)	C1—C2	1.534 (6)
N1—C3	1.482 (3)	C2—Cl3	1.776 (3)
N1—C4	1.483 (3)		
			
Cl1^i^—Co1—Cl1	111.86 (3)	C3—N1—C1	105.4 (3)
Cl1—Co1—Cl2	108.17 (2)	C4—N1—C1	114.8 (4)
Cl1—Co1—Cl2^i^	104.58 (2)	N1—C1—C2	116.2 (5)
Cl2^i^—Co1—Cl2	119.62 (4)	C1—C2—Cl3	111.0 (4)
C3—N1—C4	110.6 (2)		

Symmetry code: (i) 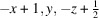 .

**Table 2:** Selected geometric parameters (Å, °) for zincate[Chem scheme1]

Zn1—Cl2	2.2553 (5)	N1—C4	1.485 (3)
Zn1—Cl1	2.2883 (5)	N1—C1	1.515 (5)
N1—C1*A*	1.476 (9)	C1—C2	1.535 (5)
N1—C3	1.480 (3)	C2—Cl3	1.779 (3)
			
Cl2—Zn1—Cl2^i^	118.93 (3)	C3—N1—C4	110.5 (2)
Cl2—Zn1—Cl1^i^	104.94 (2)	C3—N1—C1	105.3 (2)
Cl2—Zn1—Cl1	108.36 (2)	C4—N1—C1	114.6 (3)
Cl1^i^—Zn1—Cl1	111.38 (3)	N1—C1—C2	116.3 (4)
C1*A*—N1—C3	119.8 (5)	C1—C2—Cl3	110.7 (3)
C1*A*—N1—C4	111.1 (8)		

Symmetry code: (i) 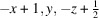 .

**Table 3:** Hydrogen-bond geometry (Å, °) for cobaltate[Chem scheme1]

*D*—H⋯*A*	*D*—H	H⋯*A*	*D*⋯*A*	*D*—H⋯*A*
N1—H1⋯Cl1	0.87 (3)	2.51 (3)	3.3093 (19)	152 (2)
N1—H1⋯Cl2^i^	0.87 (3)	3.02 (3)	3.564 (2)	122 (2)

Symmetry code: (i) 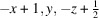 .

**Table 4:** Hydrogen-bond geometry (Å, °) for zincate[Chem scheme1]

*D*—H⋯*A*	*D*—H	H⋯*A*	*D*⋯*A*	*D*—H⋯*A*
N1—H1⋯Cl1^i^	0.87 (3)	2.51 (3)	3.3124 (18)	156 (2)
N1—H1⋯Cl2	0.87 (3)	3.03 (3)	3.5620 (19)	121 (2)

Symmetry code: (i) 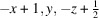 .

**Table 5:** Experimental details

	Cobaltate	Zincate
Crystal data		
Chemical formula	(C_4_H_11_ClN)_2_[CoCl_4_]	(C_4_H_11_ClN)_2_[ZnCl_4_]
*M*_r_	417.93	424.34
Crystal system, space group	Monoclinic, *C*2/*c*	Monoclinic, *C*2/*c*
Temperature (K)	295	295
*a*, *b*, *c* (Å)	12.7521 (6), 8.9648 (4), 16.6801 (10)	12.7297 (7), 8.9784 (5), 16.6837 (11)
β (°)	111.057 (1)	111.062 (2)
*V* (Å^3^)	1779.53 (16)	1779.43 (18)
*Z*	4	4
Radiation type	Mo *K*α	Mo *K*α
μ (mm^−1^)	1.85	2.26
Crystal size (mm)	0.30 × 0.27 × 0.26	0.39 × 0.38 × 0.29

Data collection		
Diffractometer	Bruker D8 Quest Eco	Bruker D8 Quest Eco
Absorption correction	Multi-scan (*SADABS*; Krause *et al.*, 2015 ▸)	Multi-scan (*SADABS*; Krause *et al.*, 2015 ▸)
*T*_min_, *T*_max_	0.696, 0.746	0.472, 0.560
No. of measured, independent and observed [*I* > 2σ(*I*)] reflections	23748, 2041, 1676	46381, 2843, 2479
*R*_int_	0.042	0.036
(sin θ/λ)_max_ (Å^−1^)	0.650	0.725

Refinement		
*R*[*F* ^2^ > 2σ(*F* ^2^)], *wR*(*F* ^2^), *S*	0.030, 0.064, 1.09	0.033, 0.070, 1.12
No. of reflections	2041	2843
No. of parameters	113	113
No. of restraints	1	1
H-atom treatment	H atoms treated by a mixture of independent and constrained refinement	H atoms treated by a mixture of independent and constrained refinement
Δρ_max_, Δρ_min_ (e Å^−3^)	0.38, −0.29	0.57, −0.40

Computer programs: *APEX3* and *SAINT* (Bruker, 2017 ▸), *SHELXT2018/2* (Sheldrick, 2015*a* ▸), *SHELXL2018/3* (Sheldrick 2015*b* ▸), *ORTEP-3 for Windows* (Farrugia, 2012 ▸) and *publCIF* (Westrip, 2010 ▸).

## supplementary crystallographic information

### Bis(2-chloro-N,N-dimethylethan-1-aminium) tetrachloridocobaltate(II) (cobaltate) Crystal data

**Table:**

(C_4_H_11_ClN)_2_[CoCl_4_]	*F*(000) = 852
*M**_r_* = 417.93	*D*_x_ = 1.56 Mg m^−^^3^
Monoclinic, *C*2/*c*	Mo *K*α radiation, λ = 0.71073 Å
Hall symbol: -C 2yc	Cell parameters from 9986 reflections
*a* = 12.7521 (6) Å	θ = 2.9–27.4°
*b* = 8.9648 (4) Å	µ = 1.85 mm^−^^1^
*c* = 16.6801 (10) Å	*T* = 295 K
β = 111.057 (1)°	Gem, blue
*V* = 1779.53 (16) Å^3^	0.30 × 0.27 × 0.26 mm
*Z* = 4	

### Bis(2-chloro-N,N-dimethylethan-1-aminium) tetrachloridocobaltate(II) (cobaltate) Data collection

**Table:**

Bruker D8 Quest Eco diffractometer	1676 reflections with *I* > 2σ(*I*)
φ and ω scans	*R*_int_ = 0.042
Absorption correction: multi-scan (SADABS; Krause *et al.*, 2015)	θ_max_ = 27.5°, θ_min_ = 3.4°
*T*_min_ = 0.696, *T*_max_ = 0.746	*h* = −16→16
23748 measured reflections	*k* = −11→11
2041 independent reflections	*l* = −21→21

### Bis(2-chloro-N,N-dimethylethan-1-aminium) tetrachloridocobaltate(II) (cobaltate) Refinement

**Table:**

Refinement on *F*^2^	Hydrogen site location: mixed
Least-squares matrix: full	H atoms treated by a mixture of independent and constrained refinement
*R*[*F*^2^ > 2σ(*F*^2^)] = 0.030	*w* = 1/[σ^2^(*F*_o_^2^) + (0.0202*P*)^2^ + 2.2595*P*] where *P* = (*F*_o_^2^ + 2*F*_c_^2^)/3
*wR*(*F*^2^) = 0.064	(Δ/σ)_max_ = 0.001
*S* = 1.09	Δρ_max_ = 0.38 e Å^−^^3^
2041 reflections	Δρ_min_ = −0.29 e Å^−^^3^
113 parameters	Extinction correction: *SHELXL2018/3* (Sheldrick 2015b)
1 restraint	Extinction coefficient: 0.0038 (3)
Primary atom site location: dual	

### Bis(2-chloro-N,N-dimethylethan-1-aminium) tetrachloridocobaltate(II) (cobaltate) Special details

**Table:**

Geometry. All esds (except the esd in the dihedral angle between two l.s. planes) are estimated using the full covariance matrix. The cell esds are taken into account individually in the estimation of esds in distances, angles and torsion angles; correlations between esds in cell parameters are only used when they are defined by crystal symmetry. An approximate (isotropic) treatment of cell esds is used for estimating esds involving l.s. planes.

### Bis(2-chloro-N,N-dimethylethan-1-aminium) tetrachloridocobaltate(II) (cobaltate) Fractional atomic coordinates and isotropic or equivalent isotropic displacement parameters (Å^2^)

**Table:**

	*x*	*y*	*z*	*U*_iso_*/*U*_eq_	Occ. (<1)
Co1	0.5	0.77754 (4)	0.25	0.03399 (13)	
Cl1	0.63314 (4)	0.92047 (6)	0.34886 (3)	0.04269 (15)	
Cl2	0.40423 (5)	0.65066 (7)	0.31892 (4)	0.05376 (18)	
N1	0.84438 (16)	0.6926 (2)	0.36634 (12)	0.0402 (4)	
H1	0.775 (2)	0.725 (3)	0.3517 (17)	0.066 (8)*	
C1	0.8515 (7)	0.5245 (5)	0.3673 (6)	0.0395 (13)	0.707 (2)
H1A	0.929586	0.496545	0.381431	0.047*	0.707 (2)
H1B	0.810217	0.488369	0.309775	0.047*	0.707 (2)
C2	0.8069 (3)	0.4440 (4)	0.4297 (2)	0.0465 (8)	0.707 (2)
H2A	0.848364	0.477204	0.487909	0.056*	0.707 (2)
H2B	0.818475	0.337488	0.427118	0.056*	0.707 (2)
Cl3	0.66156 (8)	0.48055 (11)	0.40385 (6)	0.0581 (3)	0.707 (2)
C1A	0.8273 (19)	0.5310 (12)	0.3788 (17)	0.055 (5)	0.293 (2)
H1AA	0.800336	0.479291	0.324113	0.066*	0.293 (2)
H1AB	0.897006	0.48509	0.415285	0.066*	0.293 (2)
C2A	0.7424 (9)	0.5258 (10)	0.4203 (6)	0.051 (2)	0.293 (2)
H2AA	0.672082	0.570241	0.383851	0.061*	0.293 (2)
H2AB	0.768918	0.576965	0.475201	0.061*	0.293 (2)
Cl3A	0.7270 (3)	0.3331 (3)	0.43356 (19)	0.0799 (11)	0.293 (2)
C3	0.8885 (2)	0.7413 (3)	0.29966 (17)	0.0572 (7)	
H3A	0.885462	0.848115	0.295397	0.086*	
H3B	0.843773	0.698591	0.245341	0.086*	
H3C	0.964987	0.708767	0.315027	0.086*	
C4	0.9070 (3)	0.7654 (3)	0.44973 (17)	0.0687 (8)	
H4A	0.984779	0.737603	0.468255	0.103*	
H4B	0.876356	0.733971	0.491816	0.103*	
H4C	0.900302	0.871732	0.443083	0.103*	

### Bis(2-chloro-N,N-dimethylethan-1-aminium) tetrachloridocobaltate(II) (cobaltate) Atomic displacement parameters (Å^2^)

**Table:**

	*U*^11^	*U*^22^	*U*^33^	*U*^12^	*U*^13^	*U*^23^
Co1	0.0301 (2)	0.0362 (2)	0.0354 (2)	0	0.01147 (16)	0
Cl1	0.0383 (3)	0.0475 (3)	0.0375 (3)	−0.0055 (2)	0.0078 (2)	−0.0033 (2)
Cl2	0.0611 (4)	0.0447 (3)	0.0666 (4)	−0.0097 (3)	0.0364 (3)	0.0013 (3)
N1	0.0339 (9)	0.0456 (11)	0.0413 (10)	0.0079 (8)	0.0137 (8)	−0.0021 (8)
C1	0.043 (4)	0.040 (2)	0.042 (3)	−0.0033 (16)	0.023 (2)	0.0027 (16)
C2	0.0492 (19)	0.0449 (19)	0.0467 (19)	0.0064 (16)	0.0188 (15)	0.0019 (16)
Cl3	0.0479 (6)	0.0665 (6)	0.0663 (6)	−0.0018 (4)	0.0280 (4)	−0.0007 (5)
C1A	0.038 (9)	0.086 (10)	0.047 (9)	−0.015 (6)	0.024 (5)	0.001 (6)
C2A	0.062 (6)	0.047 (5)	0.048 (5)	0.004 (5)	0.025 (4)	0.009 (4)
Cl3A	0.113 (2)	0.0584 (16)	0.0850 (19)	−0.0153 (14)	0.0554 (17)	0.0128 (13)
C3	0.0704 (17)	0.0520 (15)	0.0552 (15)	−0.0085 (13)	0.0297 (13)	0.0012 (12)
C4	0.081 (2)	0.0703 (19)	0.0485 (15)	0.0016 (15)	0.0150 (14)	−0.0161 (14)

### Bis(2-chloro-N,N-dimethylethan-1-aminium) tetrachloridocobaltate(II) (cobaltate) Geometric parameters (Å, º)

**Table:**

Co1—Cl1	2.2873 (6)	C1—H1B	0.97
Co1—Cl2	2.2618 (6)	C1—C2	1.534 (6)
N1—C3	1.482 (3)	C2—H2A	0.97
C3—H3A	0.96	C2—H2B	0.97
C3—H3B	0.96	C2—Cl3	1.776 (3)
C3—H3C	0.96	N1—C1A	1.490 (10)
N1—C4	1.483 (3)	C1A—H1AA	0.97
C4—H4A	0.96	C1A—H1AB	0.97
C4—H4B	0.96	C1A—C2A	1.480 (15)
C4—H4C	0.96	C2A—H2AA	0.97
N1—H1	0.87 (3)	C2A—H2AB	0.97
N1—C1	1.509 (5)	C2A—Cl3A	1.762 (9)
C1—H1A	0.97		
			
Cl1^i^—Co1—Cl1	111.86 (3)	C2—C1—H1A	108.2
Cl1—Co1—Cl2	108.17 (2)	N1—C1—H1B	108.2
Cl1—Co1—Cl2^i^	104.58 (2)	C2—C1—H1B	108.2
Cl2^i^—Co1—Cl2	119.62 (4)	H1A—C1—H1B	107.4
C3—N1—C4	110.6 (2)	C1—C2—Cl3	111.0 (4)
N1—C3—H3A	109.5	C1—C2—H2A	109.4
N1—C3—H3B	109.5	Cl3—C2—H2A	109.4
H3A—C3—H3B	109.5	C1—C2—H2B	109.4
N1—C3—H3C	109.5	Cl3—C2—H2B	109.4
H3A—C3—H3C	109.5	H2A—C2—H2B	108
H3B—C3—H3C	109.5	C3—N1—C1A	120.4 (7)
N1—C4—H4A	109.5	C4—N1—C1A	110.7 (11)
N1—C4—H4B	109.5	C2A—C1A—N1	105.3 (9)
H4A—C4—H4B	109.5	C2A—C1A—H1AA	110.7
N1—C4—H4C	109.5	N1—C1A—H1AA	110.7
H4A—C4—H4C	109.5	C2A—C1A—H1AB	110.7
H4B—C4—H4C	109.5	N1—C1A—H1AB	110.7
C3—N1—C1	105.4 (3)	H1AA—C1A—H1AB	108.8
C4—N1—C1	114.8 (4)	C1A—C2A—Cl3A	102.9 (7)
C3—N1—H1	108.0 (18)	C1A—C2A—H2AA	111.2
C4—N1—H1	105.3 (18)	Cl3A—C2A—H2AA	111.2
C1A—N1—H1	100 (2)	C1A—C2A—H2AB	111.2
C1—N1—H1	112.6 (19)	Cl3A—C2A—H2AB	111.2
N1—C1—C2	116.2 (5)	H2AA—C2A—H2AB	109.1
N1—C1—H1A	108.2		
			
C3—N1—C1—C2	−178.1 (5)	C3—N1—C1A—C2A	−156.9 (10)
C4—N1—C1—C2	59.9 (7)	C4—N1—C1A—C2A	71.9 (16)
N1—C1—C2—Cl3	61.6 (7)	N1—C1A—C2A—Cl3A	−179.7 (13)

Symmetry code: (i) −*x*+1, *y*, −*z*+1/2.

### Bis(2-chloro-N,N-dimethylethan-1-aminium) tetrachloridocobaltate(II) (cobaltate) Hydrogen-bond geometry (Å, º)

**Table:**

*D*—H···*A*	*D*—H	H···*A*	*D*···*A*	*D*—H···*A*
N1—H1···Cl1	0.87 (3)	2.51 (3)	3.3093 (19)	152 (2)
N1—H1···Cl2^i^	0.87 (3)	3.02 (3)	3.564 (2)	122 (2)

Symmetry code: (i) −*x*+1, *y*, −*z*+1/2.

### Bis(2-chloro-N,N-dimethylethan-1-aminium) tetrachloridozincate(II) (zincate) Crystal data

**Table:**

(C_4_H_11_ClN)_2_[ZnCl_4_]	*F*(000) = 864
*M**_r_* = 424.34	*D*_x_ = 1.584 Mg m^−^^3^
Monoclinic, *C*2/*c*	Mo *K*α radiation, λ = 0.71073 Å
Hall symbol: -C 2yc	Cell parameters from 9979 reflections
*a* = 12.7297 (7) Å	θ = 2.8–33.7°
*b* = 8.9784 (5) Å	µ = 2.26 mm^−^^1^
*c* = 16.6837 (11) Å	*T* = 295 K
β = 111.062 (2)°	Irregular, colourless
*V* = 1779.43 (18) Å^3^	0.39 × 0.38 × 0.29 mm
*Z* = 4	

### Bis(2-chloro-N,N-dimethylethan-1-aminium) tetrachloridozincate(II) (zincate) Data collection

**Table:**

Bruker D8 Quest Eco diffractometer	2479 reflections with *I* > 2σ(*I*)
φ and ω scans	*R*_int_ = 0.036
Absorption correction: multi-scan (SADABS; Krause *et al.*, 2015)	θ_max_ = 31.0°, θ_min_ = 3.4°
*T*_min_ = 0.472, *T*_max_ = 0.560	*h* = −18→18
46381 measured reflections	*k* = −12→12
2843 independent reflections	*l* = −24→24

### Bis(2-chloro-N,N-dimethylethan-1-aminium) tetrachloridozincate(II) (zincate) Refinement

**Table:**

Refinement on *F*^2^	Hydrogen site location: mixed
Least-squares matrix: full	H atoms treated by a mixture of independent and constrained refinement
*R*[*F*^2^ > 2σ(*F*^2^)] = 0.033	*w* = 1/[σ^2^(*F*_o_^2^) + (0.0155*P*)^2^ + 3.2372*P*] where *P* = (*F*_o_^2^ + 2*F*_c_^2^)/3
*wR*(*F*^2^) = 0.070	(Δ/σ)_max_ = 0.001
*S* = 1.12	Δρ_max_ = 0.57 e Å^−^^3^
2843 reflections	Δρ_min_ = −0.40 e Å^−^^3^
113 parameters	Extinction correction: *SHELXL2018/3* (Sheldrick 2015b)
1 restraint	Extinction coefficient: 0.0035 (3)
Primary atom site location: dual	

### Bis(2-chloro-N,N-dimethylethan-1-aminium) tetrachloridozincate(II) (zincate) Special details

**Table:**

Geometry. All esds (except the esd in the dihedral angle between two l.s. planes) are estimated using the full covariance matrix. The cell esds are taken into account individually in the estimation of esds in distances, angles and torsion angles; correlations between esds in cell parameters are only used when they are defined by crystal symmetry. An approximate (isotropic) treatment of cell esds is used for estimating esds involving l.s. planes.

### Bis(2-chloro-N,N-dimethylethan-1-aminium) tetrachloridozincate(II) (zincate) Fractional atomic coordinates and isotropic or equivalent isotropic displacement parameters (Å^2^)

**Table:**

	*x*	*y*	*z*	*U*_iso_*/*U*_eq_	Occ. (<1)
Zn1	0.5	0.72176 (4)	0.25	0.03333 (10)	
Cl1	0.63307 (4)	0.57810 (6)	0.34859 (3)	0.04085 (12)	
Cl2	0.40494 (5)	0.84939 (6)	0.31868 (4)	0.05219 (15)	
N1	0.15576 (15)	0.8071 (2)	0.13379 (11)	0.0396 (4)	
H1	0.223 (2)	0.770 (3)	0.1486 (17)	0.055 (7)*	
C1	0.1482 (5)	0.9756 (5)	0.1328 (5)	0.0393 (10)	0.697 (2)
H1A	0.189643	1.011624	0.190345	0.047*	0.697 (2)
H1B	0.069934	1.003059	0.118912	0.047*	0.697 (2)
C2	0.1923 (3)	1.0567 (4)	0.0703 (2)	0.0451 (7)	0.697 (2)
H2A	0.180985	1.163084	0.073337	0.054*	0.697 (2)
H2B	0.15046	1.024182	0.012066	0.054*	0.697 (2)
Cl3	0.33801 (8)	1.01959 (11)	0.09585 (6)	0.0569 (3)	0.697 (2)
C1A	0.1726 (14)	0.9672 (11)	0.1224 (12)	0.052 (4)	0.303 (2)
H1C	0.200388	1.01761	0.177512	0.063*	0.303 (2)
H1D	0.102578	1.01361	0.086803	0.063*	0.303 (2)
C2A	0.2568 (7)	0.9742 (9)	0.0801 (5)	0.0480 (18)	0.303 (2)
H2C	0.229594	0.923437	0.025068	0.058*	0.303 (2)
H2D	0.32746	0.929578	0.115896	0.058*	0.303 (2)
Cl3A	0.2728 (3)	1.1668 (3)	0.06669 (18)	0.0773 (9)	0.303 (2)
C3	0.1113 (2)	0.7582 (3)	0.20017 (16)	0.0552 (6)	
H3A	0.034943	0.791657	0.185019	0.083*	
H3B	0.156482	0.799774	0.254694	0.083*	
H3C	0.113532	0.65148	0.203863	0.083*	
C4	0.0928 (3)	0.7347 (3)	0.05022 (17)	0.0675 (8)	
H4A	0.10053	0.628507	0.056467	0.101*	
H4B	0.122638	0.767543	0.007913	0.101*	
H4C	0.014696	0.761126	0.032261	0.101*	

### Bis(2-chloro-N,N-dimethylethan-1-aminium) tetrachloridozincate(II) (zincate) Atomic displacement parameters (Å^2^)

**Table:**

	*U*^11^	*U*^22^	*U*^33^	*U*^12^	*U*^13^	*U*^23^
Zn1	0.02977 (15)	0.03496 (17)	0.03503 (17)	0	0.01136 (12)	0
Cl1	0.0366 (2)	0.0455 (3)	0.0358 (2)	0.00580 (19)	0.00738 (18)	0.00353 (19)
Cl2	0.0601 (3)	0.0429 (3)	0.0653 (4)	0.0098 (2)	0.0367 (3)	−0.0012 (2)
N1	0.0331 (8)	0.0453 (10)	0.0401 (9)	0.0091 (7)	0.0129 (7)	−0.0022 (7)
C1	0.042 (3)	0.0422 (18)	0.040 (2)	−0.0022 (15)	0.0234 (18)	0.0028 (14)
C2	0.0476 (16)	0.0459 (17)	0.0437 (16)	0.0085 (14)	0.0186 (13)	0.0044 (13)
Cl3	0.0477 (5)	0.0645 (6)	0.0649 (6)	−0.0009 (4)	0.0280 (4)	0.0002 (4)
C1A	0.042 (7)	0.083 (8)	0.043 (6)	−0.022 (5)	0.027 (4)	−0.003 (5)
C2A	0.055 (5)	0.052 (4)	0.042 (4)	0.001 (4)	0.023 (3)	0.008 (3)
Cl3A	0.110 (2)	0.0557 (13)	0.0823 (17)	−0.0148 (13)	0.0536 (16)	0.0132 (12)
C3	0.0694 (16)	0.0485 (13)	0.0532 (13)	−0.0096 (11)	0.0288 (12)	0.0008 (10)
C4	0.0780 (19)	0.0693 (18)	0.0481 (13)	0.0019 (15)	0.0139 (13)	−0.0172 (13)

### Bis(2-chloro-N,N-dimethylethan-1-aminium) tetrachloridozincate(II) (zincate) Geometric parameters (Å, º)

**Table:**

Zn1—Cl2	2.2553 (5)	C2—H2B	0.97
Zn1—Cl2^i^	2.2554 (5)	C1A—C2A	1.481 (12)
Zn1—Cl1^i^	2.2883 (5)	C1A—H1C	0.97
Zn1—Cl1	2.2883 (5)	C1A—H1D	0.97
N1—C1A	1.476 (9)	C2A—Cl3A	1.765 (8)
N1—C3	1.480 (3)	C2A—H2C	0.97
N1—C4	1.485 (3)	C2A—H2D	0.97
N1—C1	1.515 (5)	C3—H3A	0.96
N1—H1	0.87 (3)	C3—H3B	0.96
C1—C2	1.535 (5)	C3—H3C	0.96
C1—H1A	0.97	C4—H4A	0.96
C1—H1B	0.97	C4—H4B	0.96
C2—Cl3	1.779 (3)	C4—H4C	0.96
C2—H2A	0.97		
			
Cl2—Zn1—Cl2^i^	118.93 (3)	H2A—C2—H2B	108.1
Cl2—Zn1—Cl1^i^	104.94 (2)	N1—C1A—C2A	105.4 (7)
Cl2^i^—Zn1—Cl1^i^	108.36 (2)	N1—C1A—H1C	110.7
Cl2—Zn1—Cl1	108.36 (2)	C2A—C1A—H1C	110.7
Cl2^i^—Zn1—Cl1	104.94 (2)	N1—C1A—H1D	110.7
Cl1^i^—Zn1—Cl1	111.38 (3)	C2A—C1A—H1D	110.7
C1A—N1—C3	119.8 (5)	H1C—C1A—H1D	108.8
C1A—N1—C4	111.1 (8)	C1A—C2A—Cl3A	103.8 (6)
C3—N1—C4	110.5 (2)	C1A—C2A—H2C	111
C3—N1—C1	105.3 (2)	Cl3A—C2A—H2C	111
C4—N1—C1	114.6 (3)	C1A—C2A—H2D	111
C1A—N1—H1	103.7 (19)	Cl3A—C2A—H2D	111
C3—N1—H1	106.0 (18)	H2C—C2A—H2D	109
C4—N1—H1	104.1 (18)	N1—C3—H3A	109.5
C1—N1—H1	116.1 (18)	N1—C3—H3B	109.5
N1—C1—C2	116.3 (4)	H3A—C3—H3B	109.5
N1—C1—H1A	108.2	N1—C3—H3C	109.5
C2—C1—H1A	108.2	H3A—C3—H3C	109.5
N1—C1—H1B	108.2	H3B—C3—H3C	109.5
C2—C1—H1B	108.2	N1—C4—H4A	109.5
H1A—C1—H1B	107.4	N1—C4—H4B	109.5
C1—C2—Cl3	110.7 (3)	H4A—C4—H4B	109.5
C1—C2—H2A	109.5	N1—C4—H4C	109.5
Cl3—C2—H2A	109.5	H4A—C4—H4C	109.5
C1—C2—H2B	109.5	H4B—C4—H4C	109.5
Cl3—C2—H2B	109.5		
			
C3—N1—C1—C2	178.5 (4)	C3—N1—C1A—C2A	157.7 (7)
C4—N1—C1—C2	−59.9 (6)	C4—N1—C1A—C2A	−71.4 (12)
N1—C1—C2—Cl3	−61.3 (6)	N1—C1A—C2A—Cl3A	179.3 (9)

Symmetry code: (i) −*x*+1, *y*, −*z*+1/2.

### Bis(2-chloro-N,N-dimethylethan-1-aminium) tetrachloridozincate(II) (zincate) Hydrogen-bond geometry (Å, º)

**Table:**

*D*—H···*A*	*D*—H	H···*A*	*D*···*A*	*D*—H···*A*
N1—H1···Cl1^i^	0.87 (3)	2.51 (3)	3.3124 (18)	156 (2)
N1—H1···Cl2	0.87 (3)	3.03 (3)	3.5620 (19)	121 (2)

Symmetry code: (i) −*x*+1, *y*, −*z*+1/2.
